# Retraction: Influence of resources on cue preferences in mate selection

**DOI:** 10.3389/fpsyg.2024.1434216

**Published:** 2024-05-23

**Authors:** Frontiers Editorial Office

The journal retracts the September 24 2020 article cited above.

Following publication, concerns were raised regarding the scientific validity of the article. An investigation was conducted in accordance with Frontiers' policies. The authors failed to provide a satisfactory explanation and as a result, the conclusions of the article have been deemed unreliable and the article has been retracted.

This retraction was approved by the Specialty Chief Editor and Associate Editor of Frontiers in Evolutionary Psychology. The authors agree to this retraction.

